# New diagnostic criteria and severity assessment of acute cholangitis in revised Tokyo guidelines

**DOI:** 10.1007/s00534-012-0537-3

**Published:** 2012-07-24

**Authors:** Seiki Kiriyama, Tadahiro Takada, Steven M. Strasberg, Joseph S. Solomkin, Toshihiko Mayumi, Henry A. Pitt, Dirk J. Gouma, O. James Garden, Markus W. Büchler, Masamichi Yokoe, Yasutoshi Kimura, Toshio Tsuyuguchi, Takao Itoi, Masahiro Yoshida, Fumihiko Miura, Yuichi Yamashita, Kohji Okamoto, Toshifumi Gabata, Jiro Hata, Ryota Higuchi, John A. Windsor, Philippus C. Bornman, Sheung-Tat Fan, Harijt Singh, Eduardo de Santibanes, Harumi Gomi, Shinya Kusachi, Atsuhiko Murata, Xiao-Ping Chen, Palepu Jagannath, SungGyu Lee, Robert Padbury, Miin-Fu Chen

**Affiliations:** 1 Department of Gastroenterology, Ogaki Municipal Hospital, 4-86 Minaminokawa-cho, Ogaki, Gifu 503-8502 Japan; 2 Department of Surgery, Teikyo University School of Medicine, Tokyo, Japan; 3 Section of Hepatobiliary and Pancreatic Surgery, Washington University in Saint Louis School of Medicine, Saint Louis, MO USA; 4 Department of Surgery, University of Cincinnati College of Medicine, Cincinnati, OH USA; 5 Department of Emergency and Critical Care Medicine, Ichinomiya Municipal Hospital, Ichinomiya, Japan; 6 Department of Surgery, Indiana University School of Medicine, Indianapolis, IN USA; 7 Department of Surgery, Academic Medical Center, Amsterdam, The Netherlands; 8 Clinical Surgery, The University of Edinburgh, Edinburgh, UK; 9 Department of Surgery, University of Heidelberg, Heidelberg, Germany; 10 General Internal Medicine, Nagoya Daini Red Cross Hospital, Nagoya, Japan; 11 Department of Surgical Oncology and Gastroenterological Surgery, Sapporo Medical University School of Medicine, Sapporo, Japan; 12 Department of Medicine and Clinical Oncology, Graduate School of Medicine Chiba University, Chiba, Japan; 13 Department of Gastroenterology and Hepatology, Tokyo Medical University, Tokyo, Japan; 14 Clinical Research Center Kaken Hospital, International University of Health and Welfare, Ichikawa, Japan; 15 Department of Gastroenterological Surgery, Fukuoka University School of Medicine, Fukuoka, Japan; 16 Department of Surgery, Kitakyushu Municipal Yahata Hospital, Kitakyushu, Japan; 17 Department of Radiology, Kanazawa University Graduate School of Medical Science, Kanazawa, Japan; 18 Department of Endoscopy and Ultrasound, Kawasaki Medical School, Okayama, Japan; 19 Department of Surgery, Institute of Gastroenterology Tokyo Women’s Medical University, Tokyo, Japan; 20 Department of Surgery, The University of Auckland, Auckland, New Zealand; 21 Division of General Surgery, Health Sciences, University of Cape Town, Cape Town, South Africa; 22 Department of Surgery, Queen Mary Hospital, The University of Hong Kong, Hong Kong, Hong Kong; 23 Department of Hepato-Pancreato-Biliary Surgery, Hospital Selayang, Batu caves, Malaysia; 24 Department of Surgery, Hospital Italianio, University of Buenos Aires, Buenos Aires, Argentina; 25 Center for Clinical Infectious Diseases, Jichi Medical University, Tochigi, Japan; 26 Department of Surgery, Toho University Medical Center Ohashi Hospital, Tokyo, Japan; 27 Department of Preventive Medicine and Community Health, School of Medicine, University of Occupational and Environmental Health, Kitakyushu, Japan; 28 Department of Surgery, Hepatic Surgery Centre, Tongji Hospital, Tongi Medical College, Huazhong University of Science and Technology, Wuhan, China; 29 Department of Surgical Oncology, Lilavati Hospital and Research Centre, Mumbai, India; 30 HepatoBiliary Surgery and Liver Transplantation, Asan Medical Center, Ulsan University, Ulsan, Korea; 31 Division of Surgical and Specialty Services, Flinders Medical Centre, Bedford Park, Australia; 32 Chang Gung Memorial Hospital, Chang Gung University, Taoyuan, Taiwan

**Keywords:** Acute cholangitis, Biliary infection, Diagnostic criteria, Severity assessment, Charcot’s triad

## Abstract

**Background:**

The Tokyo Guidelines for the management of acute cholangitis and cholecystitis were published in 2007 (TG07) and have been widely cited in the world literature. Because of new information that has been published since 2007, we organized the Tokyo Guidelines Revision Committee to conduct a multicenter analysis to develop the updated Tokyo Guidelines (TG13).

**Methods/materials:**

We retrospectively analyzed 1,432 biliary disease cases where acute cholangitis was suspected. The cases were collected from multiple tertiary care centers in Japan. The ‘gold standard’ for acute cholangitis in this study was that one of the three following conditions was present: (1) purulent bile was observed; (2) clinical remission following bile duct drainage; or (3) remission was achieved by antibacterial therapy alone, in patients in whom the only site of infection was the biliary tree. Comparisons were made for the validity of each diagnostic criterion among TG13, TG07 and Charcot’s triad.

**Results:**

The major changes in diagnostic criteria of TG07 were re-arrangement of the diagnostic items and exclusion of abdominal pain from the diagnostic list. The sensitivity improved from 82.8 % (TG07) to 91.8 % (TG13). While the specificity was similar to TG07, the false positive rate in cases of acute cholecystitis was reduced from 15.5 to 5.9 %. The sensitivity of Charcot’s triad was only 26.4 % but the specificity was 95.6 %. However, the false positive rate in cases of acute cholecystitis was 11.9 % and not negligible. As for severity grading, Grade II (moderate) acute cholangitis is defined as being associated with any two of the significant prognostic factors which were derived from evidence presented recently in the literature. The factors chosen allow severity assessment to be performed soon after diagnosis of acute cholangitis.

**Conclusion:**

TG13 present a new standard for the diagnosis, severity grading, and management of acute cholangitis.

## Introduction

Patients with acute cholangitis are at risk for developing severe infection that can be fatal unless appropriate medical care is provided at an early stage. Advances in antibiotic therapy and acute care as well as a wide diffusion of expertise in biliary endoscopy have resulted in reduction of morbidity and mortality from acute cholangitis. However, it remains a life-threatening disease and early determination of disease severity is essential to select appropriate therapy, particularly the timing of biliary decompression. In 2007, we conducted a systematic review and sponsored an international consensus conference in Tokyo. This meeting resulted in the introduction of the new Tokyo Guidelines (TG07) for diagnosis and severity assessment of acute cholangitis [1].

Diagnostic and severity assessment criteria need to be updated periodically based on new information, criticisms, and suggestions for improvement. For instance, ever since Charcot reported a patient with severe acute cholangitis as a case of ‘hepatic fever’ in 1877, Charcot’s triad has been widely considered to be one of the most important diagnostic criteria [2–6]. However, Charcot’s triad has extremely low sensitivity despite its high specificity. In addition, false positive cases of acute cholecystitis are not unusual with this classic diagnostic triad.

With experience we and others found potential shortcomings in TG07 [7]. Consequently, the Tokyo Guidelines Revision Committee was assembled and gathered a large number of cases of acute cholangitis from tertiary care centers in Japan. These cases acted as a gold standard to assess diagnostic and severity criteria such as TG07. The present study has confirmed limitations of TG07 and presents updated TG13 criteria which have improved sensitivity and specificity and which importantly, unlike the criteria in TG07, allow severity assessment at the time of presentation so that biliary drainage or other procedures can be performed without delay.

## Methods

In the present multicenter study, 1,432 patients were enrolled with biliary tract abnormalities and suspected acute cholangitis between January 2007 and July 2011. Choledocholithisis or biliary stricture was confirmed by direct cholangiography (i.e., endoscopic retrograde cholangiopancreatography (ERCP), percutaneous transhepatic cholangiography). Acute cholecystitis was confirmed by pathologic examination of excised gallbladders.

The establishment of guidelines for diagnosis and severity assessment in a disease requires that there is diagnostic certainty by which to assess criteria. For acute cholecystitis this may be provided by pathologic examination of excised gallbladders; however, pathologic specimens are not available in acute cholangitis. Our approach in this study was to gather data from 794 patients who were considered to have had acute cholangitis based on one of the following three criteria: (1) presence of purulent biliary leakage; (2) clinical remission due to bile duct drainage; or (3) remission achieved by antimicrobial therapy alone in patients in whom the only site of infection was the biliary tree. For comparison we also gathered data from 638 patients who had other biliary tract abnormalities (Table 1).

**Table 1:** Clinical characteristics of patients

	Acute cholangitis (*n* = 794)^a^	Other disease (*n* = 638)
	Etiology; choledocholithiasis (*n* = 402) malignant tumor (*n* = 392)	Choledocholithiasis (*n* = 178), obstructive jaundice caused by malignant tumor (*n* = 241) acute cholecystitis (*n* = 219)
Age	71.7 ± 11.8	68.5 ± 12.3
Sex (male:female)	490:304	307:331
Charcot triad	147 (18.5 %)	26 (4.1 %)
Abdominal pain	435 (54.8 %)	309 (48.4 %)

Presence of purulent biliary leakage

Clinical remission due to bile duct drainage

Remission achieved by antimicrobial therapy alone in patients in whom the only site of infection was the biliary tree

^a^The ‘Gold Standard’ for acute cholangitis in this study was that one of the following three conditions was present

Using these patients, we adjusted diagnostic criteria to have the highest sensitivity and specificity for acute cholangitis. For establishment of new severity assessment criteria, we examined variables reported in the literature either as predictive of poor prognosis in acute cholangitis or of need for urgent biliary drainage (Table 2). These variables were then used to construct a grading system that would permit determination of the level of severity at the time of diagnosis so that those patients who need urgent biliary decompression could receive treatment without delay.

**Table 2:** Prognostic factors in acute cholangitis

Prognostic factor	Positive value	References
Hyperbilirubinemia	>2 mg/dL	[8]
	>2.2 mg/dL	[9]
	>2.93 mg/dL	[10]
	>4 mg/dL	[11, 12]
	>5.26 mg/dL	[13]
	>5.56 mg/dL	[14]
	>8.1 mg/dL, >9.2 mg/dL	[15]
	>9.1 mg/dL	[16]
	>10 mg/dL	[17]
Hypoalbuminemia	<3.0 g/dL	[10, 13, 18]
Acute renal failure	BUN (>20–>64 mg/dL) Creatinine (>1.5–>2.0 mg/dL)	[8, 9, 11, 19, 20]
Shock		[8, 12, 13, 19]
Reduced platelet count	<1,00,000–<1,50,000/mm^3^	[13, 18, 20]
Endotoxemia/bacteremia		[9, 10, 14, 20]
High fever	>38 °C	[8]
	>39 °C	[12]
	>40 °C	[16]
Medical comorbidity		[8, 11, 13, 18, 19]
Elderly patient	≥50 years old	[11]
	≥60 years old	[8]
	≥70 years old	[19, 21]
	≥75 years old	[22]
Malignancy as etiology		[9, 11, 14]
Prolonged prothrombin time	≤14 s	[10, 22]
	≤15 s	[8]
Leukocytosis	≤12,000	[8]
	≤20,000	[16, 17]
Current smoking	Yes	[21, 22]

For confirming the advantage of these revisions, updated diagnostic criteria and severity assessment criteria also were retrospectively assessed by the present multicenter analysis.

## Results

### Formulation of new diagnostic criteria for acute cholangitis

#### Assessment of Charcot’s triad and TG07 diagnostic criteria for acute cholangitis

Analysis of the 1,432 cases of biliary tract diseases showed that Charcot’s triad had low sensitivity (26.4 %) but high specificity (95.9 %) for acute cholangitis, with 11.9 % of cases of acute cholecystitis demonstrating Charcot’s triad. On the other hand, the sensitivity and specificity of TG07 diagnostic criteria were 82.6 and 79.8 %, respectively, while 11.9 % of cases acute cholecystitis would have fit the diagnostic criteria for acute cholangitis if TG07 criteria were applied (Table 3). Furthermore, TG07 diagnostic criteria for acute cholangitis were found to have insufficient sensitivity for making an early diagnosis of life-threatening acute cholangitis.

**Table 3:** Retrospective comparison of various diagnostic criteria of acute cholangitis in a multicenter study in Japan

	Charcot’s triad (%)	TG07 (%)	The first draft criteria (with abdominal pain and history of biliary disease) (%)	TG13 (%)
Sensitivity	26.4	82.6	95.1	91.8
Specificity	95.9	79.8	66.3	77.7
Positive rate in acute cholecystitis	11.9	15.5	38.8	5.9

#### Revision of TG07 diagnostic criteria for acute cholangitis

It seemed that the shortcomings of TG07 might be related to inappropriate combination of such items as clinical context and manifestations, laboratory data and imaging findings. Therefore, for TG13, categories of diagnostic items were constructed based on the three main clinical manifestations used in the diagnosis of acute cholangitis: (a) fever and/or evidence of inflammatory response, (b) jaundice and abnormal liver function tests, and (c) abdominal pain, a history of biliary diseases, biliary dilatation, or other biliary manifestations. The presence of a finding in all three of these categories has been considered to be diagnostic of acute cholangitis.

Abdominal pain and a history of biliary tract disease, however, are also common indicators of other biliary problems such as acute cholecystitis and even acute hepatitis. Acute cholecystitis application of the first draft criteria of TG13 (which included abdominal pain and a history of biliary tract disease) to patients with acute cholecystitis resulted in 38.8 % of patients with acute cholecystitis meeting the criteria for diagnosis of acute cholangitis. However, despite a high sensitivity for acute cholangitis of 95.1 % for these diagnostic criteria the specificity of (66.3 %) was disappointingly low (Table 3). In the next iteration of the diagnostic criteria, abdominal pain and the history of biliary diseases were deleted from the diagnostic criteria. This resulted in the best outcome in terms of high sensitivity and specificity for acute cholangitis and low false positive rate for acute cholecystitis (Table 3) and these were the diagnostic criteria which were adopted for TG13 (Table 3).

The final TG13 diagnostic criteria are shown in Table 4. To make a definitive diagnosis one item from each of the three categories (A–C) is required. Furthermore, a ‘suspected’ diagnosis can be made when there is one item present from the A list and one item from either the B or C list. By establishing ‘suspected diagnosis’, early biliary drainage or source control of infection among patients with acute cholangitis can be provided without waiting for a definitive diagnosis.

**Table 4:** TG13 Diagnostic criteria for acute cholangitis

A. Systemic inflammation				
A-1. Fever and/or shaking chills				
A-2. Laboratory data: evidence of inflammatory response				
B. Cholestasis				
B-1. Jaundice				
B-2. Laboratory data: abnormal liver function tests				
C. Imaging				
C-1. Biliary dilatation C-2. Evidence of the etiology on imaging (stricture, stone, stent, etc.)				
Suspected diagnosis: one item in A + one item in either B or C				
Definite diagnosis: one item in A, one item in B and one item in C				
A-2 Abnormal white blood cell counts, increase of serum C-reactive protein levels, and other changes indicating inflammation				
B-2 Increased serum ALP, r-GTP (GGT), AST, and ALT levels				
Threshholds				
	A-1	Fever		BT >38 °C
	A-2	Evidence of inflammatory response	WBC (×1,000/μL)	<4, or >10
			CRP (mg/dL)	≧1
	B-1	Jaundice		T-Bil ≧2 (mg/dL)
	B-2	Abnormal liver function tests	ALP (IU)	>1.5 × STD*
			γGTP (IU)	>1.5 × STD*
			AST (IU)	>1.5 × STD*
			ALT (IU)	>1.5 × STD*

Other factors which are helpful in diagnosis of acute cholangitis include abdominal pain (Right upper quadrant (RUQ) or upper abdominal) and a history of biliary disease such as gallstones, previous biliary procedures, and placement of a biliary stent

In acute hepatitis, marked systematic inflammatory response is observed infrequently. Virological and serological tests are required when differential diagnosis is difficult

ALP Alkaline phosphatase, r-GTP (GGT) r-glutamyltransferase,

AST aspartate aminotransferase, ALT alanine aminotransferase

* *STD* upper limit of normal value

One of the items in category A involves determination of the presence of abnormal laboratory tests. Thresholds for declaring positivity test might be set at the upper limit of normal for the tests. The disadvantage of this approach is that minor abnormalities in the tests are not uncommon in acute cholecystitis. Therefore, a somewhat higher threshold for acute cholangitis is desirable. The normal upper limit range of the liver function tests differs from facility to facility. Therefore, a fixed threshold is not practical. Instead, the threshold was set at 1.5 times the upper limit of normal in a facility. We then conducted a multicenter analysis to compare this threshold with two other types of threshold in terms of the diagnostic ability for acute cholangitis. When the threshold was set at 1.5 times the upper limit, both sensitivity and specificity were similar to those at which another two types of threshold were applied (Table 5). From the above results, it was considered appropriate and practical that the threshold was set at 1.5 times the normal upper limit for the liver function test in the particular facility.

**Table 5:** Comparisons of various cut-offs for laboratory testing results for the diagnosis of acute cholangitis in Japan

		Thresholds for positivity of test		
		Adoption	Limit of this test (low)	Limit of this test (high)
	T-Bil (mg/dL)	≧2	Same	Same
	ALP (IU)	>1.5 × STD	≧400	≧500
	γGTP (IU)	>1.5 × STD	≧100	≧150
	AST (IU)	>1.5 × STD	≧50	≧100
	ALT (IU)	>1.5 × STD	≧50	≧100
	WBC (×1,000/μL)	<4, or >10	Same	Same
	CRP (mg/dL)	≧1	Same	Same
	BT	>38 °C	Same	Same
Sensitivity		91.8 %	93.0 %	92.7 %
Specificity		77.7 %	77.9 %	77.9 %
Positive rate in acute cholecystitis (*n* = 219)		5.9 %	9.1 %	8.7 %

*STD* upper limit of normal value

### Formulation of new severity assessment criteria for acute cholangitis

#### Assessment of TG07 severity assessment criteria for acute cholangitis

The use of TG07 severity assessment criteria in actual clinical situations has shown that use of these criteria was inefficient in separating moderate cases (Grade II) from mild cases (Grade I) at the time of initial diagnosis. In TG07, Grades II and I were only assessed after observation of the initial treatment courses. In this treatment strategy, urgent biliary drainage can be indicated for cases assessed as ‘severe’, but provision of early biliary drainage is impossible for cases as ‘moderate’. The present multicenter analysis showed that many cases (46.8 %, 258 of 551 cases) of Grade II or I underwent urgent biliary drainage in the same manner as Grade III. In these cases, differentiation between grade II and Grade I was impossible, because the definition of Grade II in TG07 was ambiguous (Table 6).

**Table 6:** Timing of biliary drainage among patients with acute cholangitis diagnosed with TG07—multicenter analysis of acute cholangitis for revision of TG07 severity criteria of acute cholangitis

Timing of drainage/treatment for etiology	Grade III	Grade II	Grade I	Total
Within 24 h	41	258 (Grade II or I)		297
24–48 h	9	54	0	63
After 48 h	20	130	12	162
Drainage (−)	2	3	96	101
Total	72 (11.6 %)	551 (88.4 %) (Grade II or I)		623

#### Revision of TG07 severity assessment criteria for acute cholangitis

Given these insufficiencies of TG07 in clinical practice, a revision was sought which might improve severity assessment strategies upon diagnosis in order to allow selection of those patients who needed immediate source control of infection. Since there had been no scientifically based definitions of ‘moderate cases’ except for the consensus-based TG07 we needed a new definition of what constituted moderate cases needing early source control in TG13.

To improve TG07 we examined items reported as predictive factors of poor prognosis among patients with acute cholangitis and factors associated with the need for urgent biliary drainage (Table 2). Furthermore, factors that endoscopic gastroenterologists value in determining the timing of biliary drainage were integrated except for the factors that define Grade III cases (severe cases). Presence or absence of endotoxemia and/or bacteremia, and malignancy as etiology cannot be assessed upon the diagnosis of acute cholangitis and were therefore not included. Medical comorbidities such as diabetes mellitus and neurological diseases were considered as severity factors; however, due to their wide disease spectrum, it was decided that it was impractical to include co-morbidity in TG13. The criteria selected for moderate severity were leukocytosis high fever, age >75 years, hyperbilirubinemia, and hypoalbuminemia. The presence of any two of the five positive criteria will classify the disease as Grade II (moderate).

The revised assessment criteria for acute cholangitis are shown in Table 7.

**Table 7:** TG13 Severity assessment criteria for acute cholangitis

Grade III (Severe) acute cholangitis	
‘Grade III’ acute cholangitis is defined as acute cholangitis that is associated with the onset of dysfunction at least in any one of the following organs/systems	
1. Cardiovascular dysfunction	Hypotension requiring dopamine ≥5 μg/kg per min, or any dose of norepinephrine
2. Neurological dysfunction	Disturbance of consciousness
3. Respiratory dysfunction	PaO_2_/FiO_2_ ratio <300
4. Renal dysfunction	Oliguria, serum creatinine >2.0 mg/dL
5. Hepatic dysfunction	PT-INR >1.5
6. Hematological dysfunction	Platelet count <1,00,000/mm^3^
Grade II (moderate) acute cholangitis	
‘Grade II’ acute cholangitis is associated with any two of the following conditions:	
1. Abnormal WBC count (>12,000/mm^3^, <4,000/mm^3^)	
2. High fever (≥39 °C)	
3. Age (≥75 years)	
4. Hyperbilirubinemia (total bilirubin ≥5 mg/dL)	
5. Hypoalbuminemia (<STD × 0.7)	
Grade I (mild) acute cholangitis	
‘Grade I’ acute cholangitis does not meet the criteria of ‘Grade III (severe)’ or ‘Grade II (moderate)’ acute cholangitis at initial diagnosis	

Early diagnosis, early biliary drainage and/or treatment for etiology, and antimicrobial administration are fundamental treatment for acute cholangitis classified not only ‘Grade III (severe)’ and ‘Grade II (moderate)’ but also Grade I (mild)

Therefore, it is recommended that patients with acute cholangitis who do not respond to the initial medical treatment (general supportive care and antimicrobial therapy) undergo early biliary drainage or treatment for etiology

*STD* lower limit of normal value

#### Assessment of TG13 severity assessment criteria for acute cholangitis

We performed a multicenter analysis using the TG13 severity assessment criteria for acute cholangitis in real clinical settings. Of the 623 cases of acute cholangitis where severity grading was retrospectively made clear, there were 72 Grade III cases (11.6 %), 216 Grade II cases (34.7 %) and 335 Grade I cases (53.8 %). Furthermore, the Grade II cases requiring urgent or early biliary drainage accounted for 46 % of the acute cholangitis cases. An examination of Grade I cases where biliary drainage had been carried out within 24 h and within 48 h found 140 cases (41.8 %) and 181 cases (54.0 %), respectively. It was surprising that so many patients with Grade I criteria had undergone biliary drainage. However, on further analysis it was found that almost all Grade I cases that had undergone early biliary drainage were due to biliary obstruction such as common duct stones. These types of interventions accounted for 135 of 140 cases (94.8 %) within 24 h and 41 cases (100 %) within 48 h, respectively. The number of Grade I cases that had undergone biliary drainage as an urgent treatment to control infection were small (Table 8).

**Table 8:** Timing of biliary drainage among patients with acute cholangitis diagnosed with TG13—multicenter analysis of acute cholangitis for revision of TG07 Severity assessment criteria for acute cholangitis

Timing of drainage/treatment for etiology	Grade III	Grade II	Grade I	Total
Within 24 h	41	116	140 (135)	297
24–48 h	9	13	41 (41)	63
After 48 h	20	48	94	162
Drainage (−)	2	39	60	101
Total	72 (11.6 %)	216 (34.7 %)	335 (53.8 %)	623

() indicates the number of cases that have early drainage and treatment of etiology

Of the 110 cases of acute cholangitis that met the Charcot’s triad, 13 cases (11.8 %) have been classified as Grade III, and 52 as Grade II (47.3 %), respectively. Furthermore, approximately 80 % (59 of 72 cases) of Grade III cases in TG13 failed to satisfy Charcot’s triad (Table 9). Charcot’s triad was not found to be associated with disease severity.

**Table 9:** TG13 Severity assessment criteria and Charcot’s triad

Severity grading of TG13	Charcot’s triad	
	Yes (*n* = 110)	No (*n* = 513)
Grade III	13 (11.8 %)	59 (11.5 %)
Grade II	52 (47.3 %)	164 (32.0 %)
Grade I	45 (40.9 %)	290 (56.5 %)

## Discussion

The main goals of diagnostic and severity assessment criteria are to allow early establishment of diagnosis and selection of the most appropriate management plan for the stage of the disease. This was attempted for acute cholangitis in TG07 where the guidelines were based on available literature and input of experts at a consensus conference held in Tokyo in 2006. At that meeting, diagnostic criteria were presented combining blood tests and diagnostic imaging together with Charcot’s triad [23]. However, there is a report showing that the sensitivity was low (63.9 %) for making a definite diagnosis of acute cholangitis [7]. It is well established that guidelines need periodic assessment and revision; however, in the case of TG07 this was particularly so because of shortcomings that became evident through application in clinical practice and as a result of new information in the literature. As in TG07, initial iterations were produced in Japan with modifications incorporating the input of experts from around the world.

A particularly vexing problem in studies of acute cholangitis is how to set a gold standard for the disease against which to compare diagnostic and severity grading criteria. Unlike diseases such as acute cholecystitis there is no organ or tissue with which absolute diagnosis of acute cholangitis can be achieved pathologically. Therefore, a gold standard must be set by other means. An important step in generation of TG13 was to adopt the three gold standard diagnostic criteria suggested in the literature and by experience. This then permitted the gathering of a large number of example cases by which to refine and judge the adequacy of the new criteria. While this was an arduous task it seems that the results support this approach in dealing with these issues. Another novelty in our approach is that the diagnostic criteria were not judged simply against normal individuals but included patients with other biliary tract diseases especially acute cholecystitis. This increases the robustness of the criteria as a clinical tool.

The early iterations of the diagnostic criteria for TG13 included abdominal pain and a history of biliary tract disease; however, it was found that inclusion of these criteria resulted in a schema with low specificity and a high false positive rate in cases of acute cholecystitis. When these variables were dropped the results improved dramatically. It may seem odd to have diagnostic criteria which eliminate abdominal pain as a symptom of acute cholangitis but the benefit of eliminating confusion with other biliary tract disease if pain is included outweighs any advantage of including it.

The new TG13 diagnostic criteria have fewer variables and are arranged in more logical categories. The thresholds for laboratory tests have been selected to permit worldwide use as they do not depend on absolute values but on 1.5 times the upper limit of normal of any laboratory. As such these criteria should be amenable to use on handheld devices further improving the ability to rapidly diagnose the condition.

Ideally, a definitive diagnosis should be available at the time of presentation. If the requirement for a definitive diagnosis results in delay of biliary drainage with progression to more severe stages of the disease or death under observation the purpose of a definitive diagnosis is subverted. At the present state of knowledge our data suggest that the decision to proceed to early biliary drainage can and should be made on suspected diagnosis and severity grading as outlined in the paper as both of these can be determined at presentation. The effect of this strategy can be determined as the criteria for diagnosis are evaluated in the future.

The severity grading has also been revised based on new information available in the literature. The criteria for severe cases have not been modified but those in the important moderate group have been updated. As noted all five criteria in the moderate group are determinable at presentation. This required the exclusion of a number of criteria as outlined in the results.

In practice the diagnostic criteria and severity grading would be used in tandem at the time of presentation. If a patient fit the suspected criteria, severity grading would be performed. Those falling into the moderate and severe categories would be candidates for urgent biliary decompression, while those in the mild category would be treated initially with antibiotics. Many of the latter patients would still have biliary drainage within the first 48 h for control of the cause of acute cholangitis such as extraction of common duct stones.

A diagnosis of acute cholangitis has traditionally been made by Charcot’s triad. According to several reports, Charcot’s triad was observed for only 9 % except in cases of acute cholangitis [8], but cases of acute cholangitis presenting all of Charcot’s triad accounted for only 50–70 % [3, 8–14, 24–26]. We also continued to examine the utility of Charcot’s triad because of the prominence of this diagnostic triad in this disease. We found that Charcot’s triad shows very high specificity—the presence of the Charcot’s triad strongly suggested the presence of acute cholangitis. However, due to the low sensitivity, it is not applicable in making a diagnosis of acute cholangitis. Also as noted the triad was not associated with disease severity.

In summary TG13 presents new diagnostic and severity grading systems based on a large patients base and a reasonable gold standard. These criteria allow early diagnosis and severity grading of the disease and should be clinically useful in the management of this severe disease.

## Conclusion

TG13 introduces a new standard for the diagnosis, severity grading and management of acute cholangitis. As compared with Charcot’s triad and TG07, validity of the diagnostic criteria has been improved and severity assessment criteria have become more suitable for clinical use.
